# Supplementary material for: GSDMD-dependent neutrophil extracellular traps promote macrophage-to-myofibroblast transition and renal fibrosis in obstructive nephropathy
Source: Cell Death Dis. 2022 Aug 8;13(8):693. doi: 10.1038/s41419-022-05138-4 (PMC9360039; doi:10.1038/s41419-022-05138-4)
Supplement: Supplementary file 1 — Original Data File [file 41419_2022_5138_MOESM1_ESM.pptx]

## Slide 1
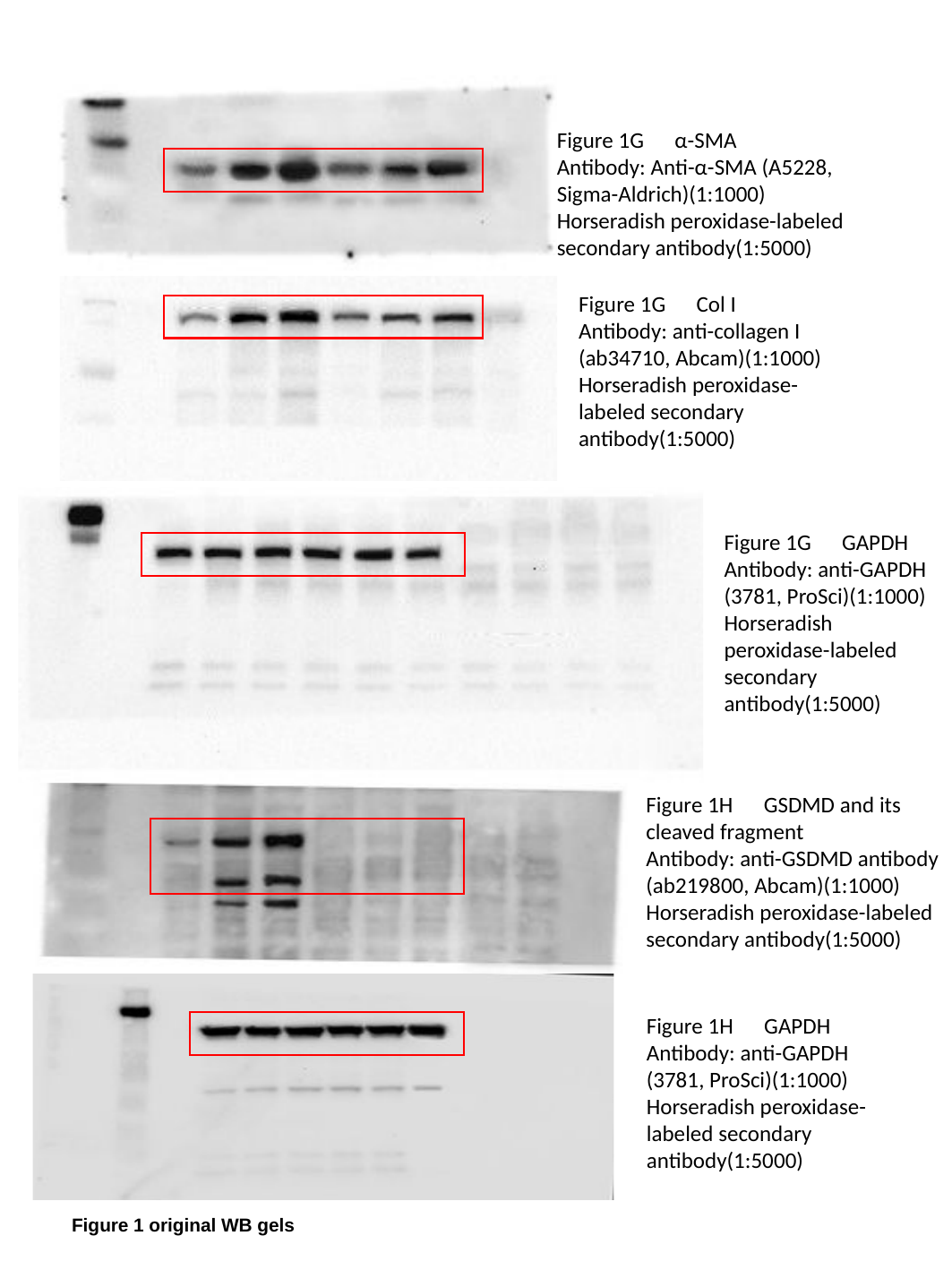

Figure 1G α-SMA
Antibody: Anti-α-SMA (A5228, Sigma-Aldrich)(1:1000)
Horseradish peroxidase-labeled secondary antibody(1:5000)
Figure 1G Col I
Antibody: anti-collagen I (ab34710, Abcam)(1:1000)
Horseradish peroxidase-labeled secondary antibody(1:5000)
Figure 1G GAPDH
Antibody: anti-GAPDH (3781, ProSci)(1:1000)
Horseradish peroxidase-labeled secondary antibody(1:5000)
Figure 1H GSDMD and its cleaved fragment
Antibody: anti-GSDMD antibody (ab219800, Abcam)(1:1000)
Horseradish peroxidase-labeled secondary antibody(1:5000)
Figure 1H GAPDH
Antibody: anti-GAPDH (3781, ProSci)(1:1000)
Horseradish peroxidase-labeled secondary antibody(1:5000)
Figure 1 original WB gels

## Slide 2
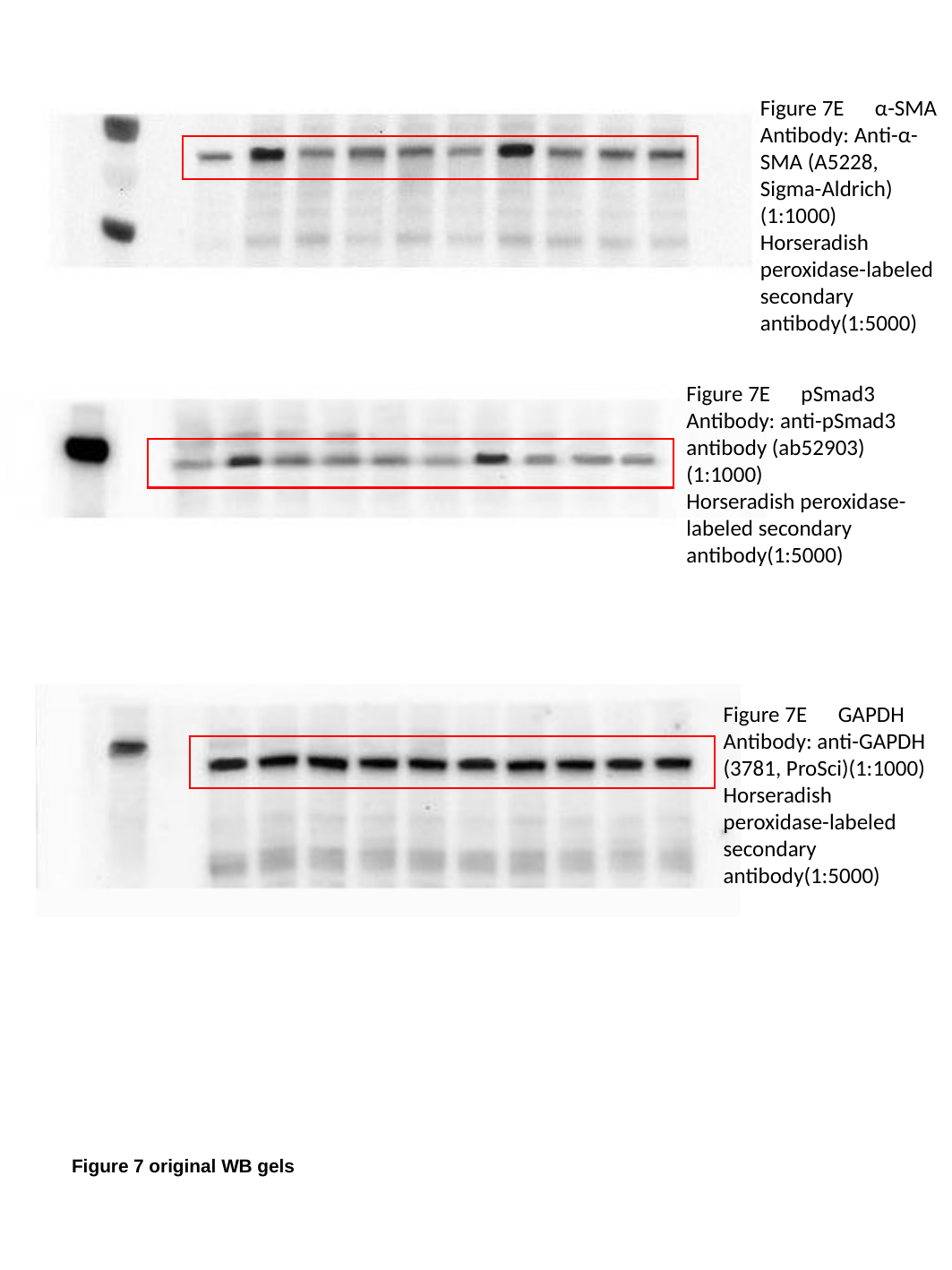

Figure 7E α-SMA
Antibody: Anti-α-SMA (A5228, Sigma-Aldrich)(1:1000)
Horseradish peroxidase-labeled secondary antibody(1:5000)
Figure 7E pSmad3
Antibody: anti-pSmad3 antibody (ab52903)(1:1000)
Horseradish peroxidase-labeled secondary antibody(1:5000)
Figure 7E GAPDH
Antibody: anti-GAPDH (3781, ProSci)(1:1000)
Horseradish peroxidase-labeled secondary antibody(1:5000)
Figure 7 original WB gels

## Slide 3
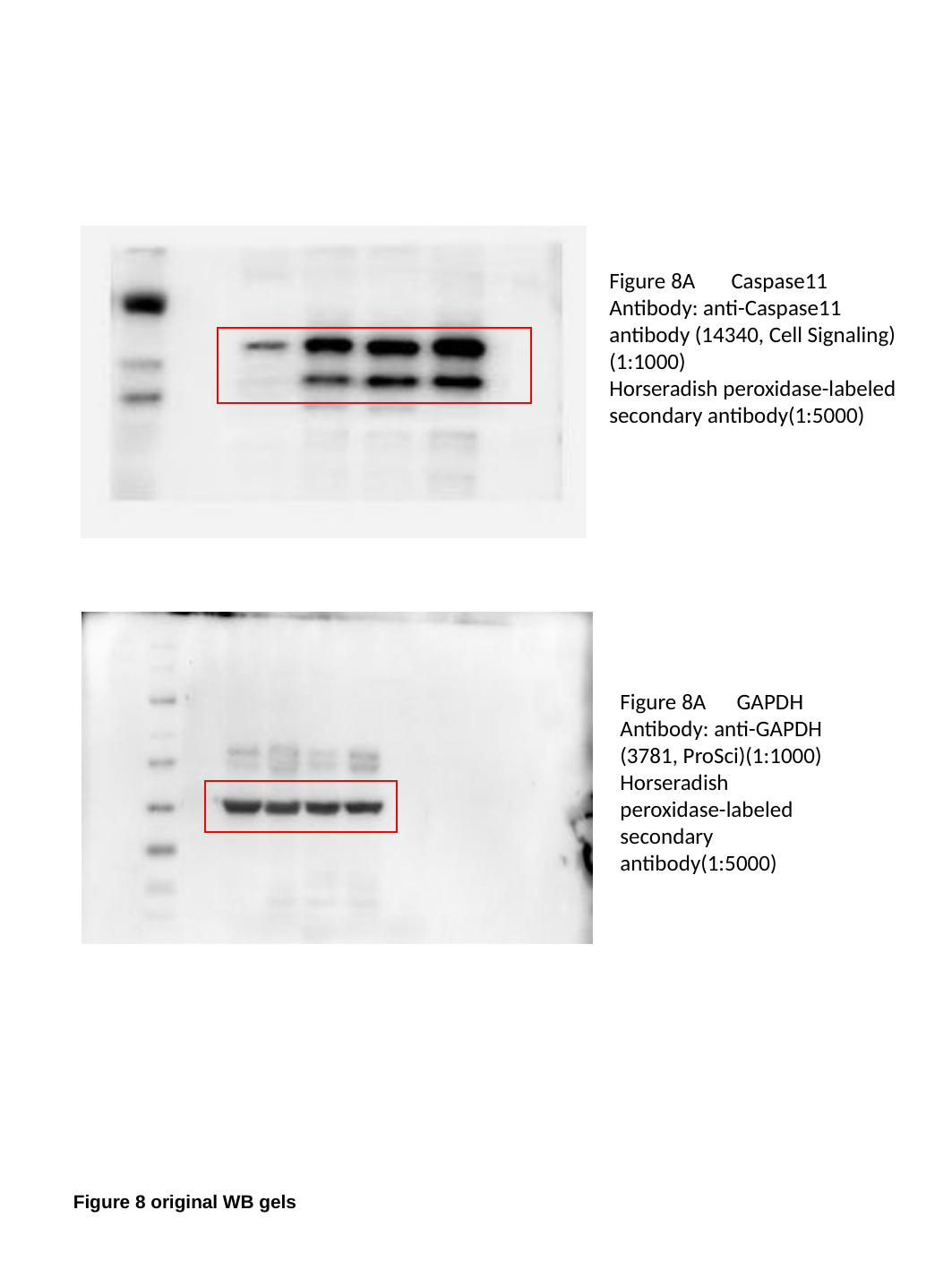

Figure 8A Caspase11
Antibody: anti-Caspase11 antibody (14340, Cell Signaling)(1:1000)
Horseradish peroxidase-labeled secondary antibody(1:5000)
Figure 8A GAPDH
Antibody: anti-GAPDH (3781, ProSci)(1:1000)
Horseradish peroxidase-labeled secondary antibody(1:5000)
Figure 8 original WB gels
