# Supplementary material for: GSDMD-dependent neutrophil extracellular traps promote macrophage-to-myofibroblast transition and renal fibrosis in obstructive nephropathy
Source: Cell Death Dis. 2022 Aug 8;13(8):693. doi: 10.1038/s41419-022-05138-4 (PMC9360039; doi:10.1038/s41419-022-05138-4)
Supplement: Supplementary file 2 — Supplemental material [file 41419_2022_5138_MOESM2_ESM.pdf]

Supplementary materials

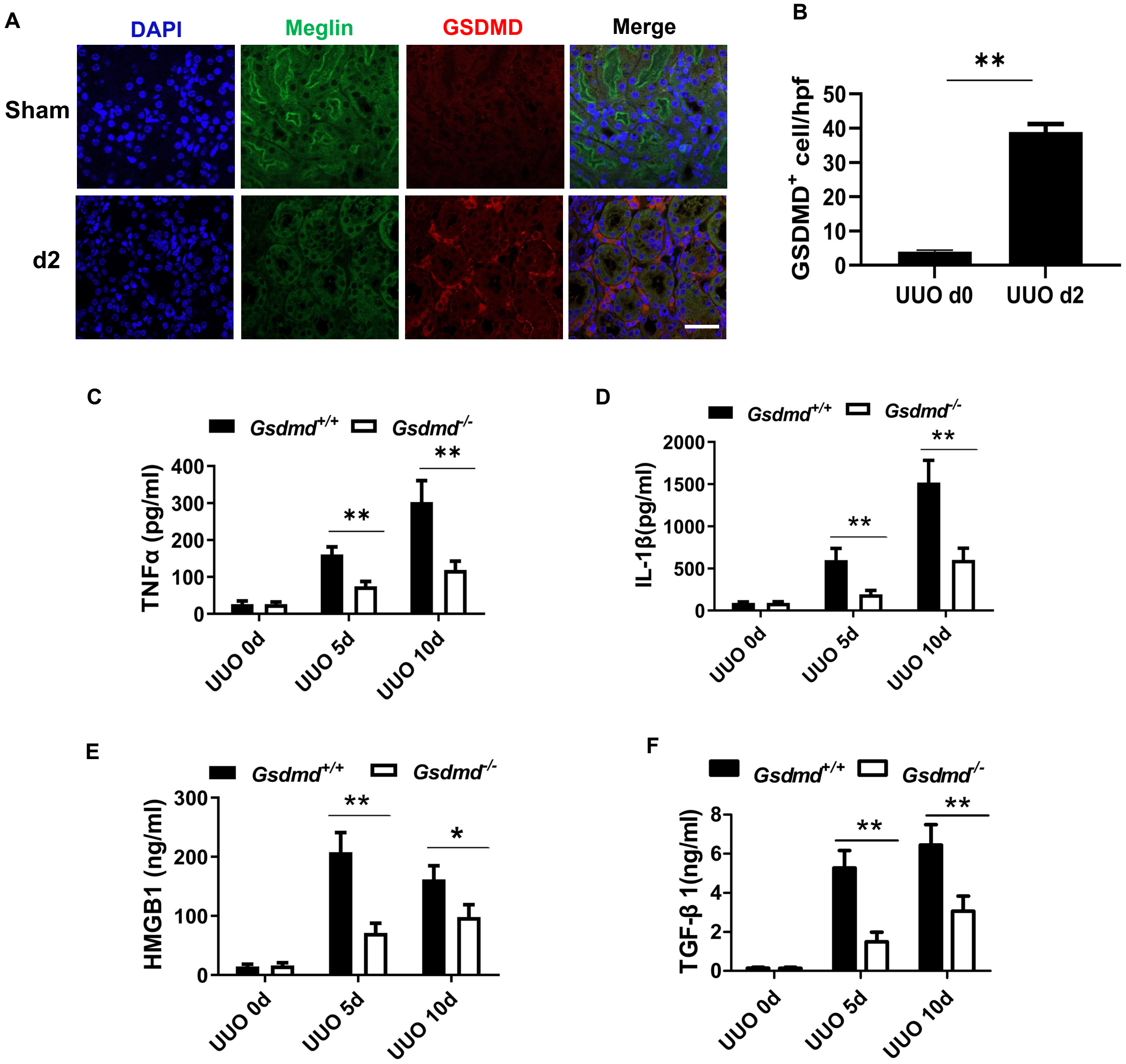

Figure S1. UUO induced GSDMD activation in the kidney and *Gsdmd* deletion inhibited inflammatory cytokines production after UUO. (A) Representative images of immunofluorescence staining of kidney sections from wild-type mice on day 0 and day 2 after UUO, showing the expression of megalin (green) and GSDMD (red). DAPI (blue) was used for nuclear staining. (B) Quantification of GSDMD-expressed cells by immunofluorescence.n=6. (C-F) TNF-α, IL-1β, HMGB1 and TGF-β production in kidney on day 0, 5 and 10 after UUO were detected by ELISA. n=5. **P* < 0.05; ***P* < 0.01.

Supplementary materials

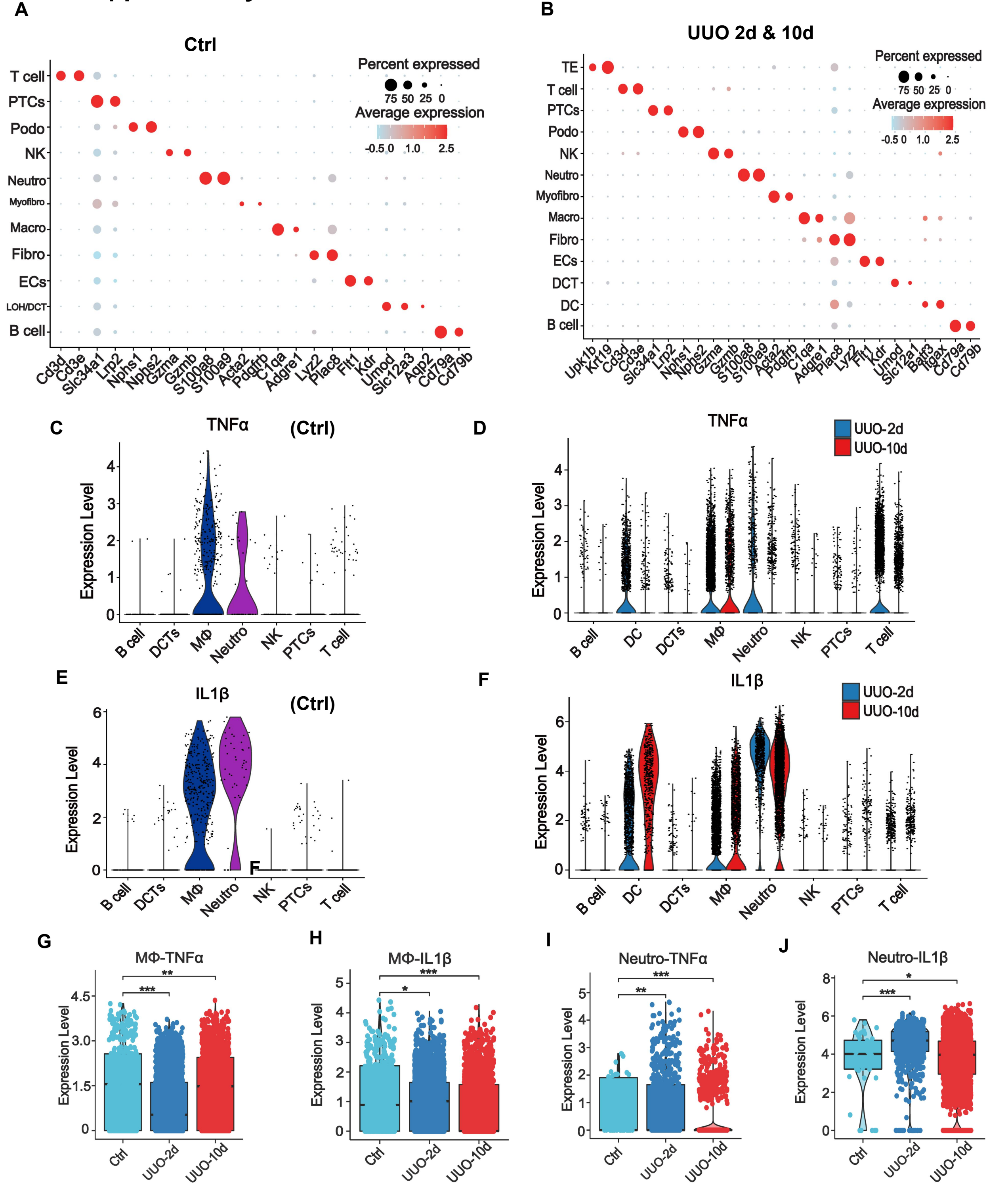

Figure S2. Single-cell sequencing showed the elevated gene expression of TNF-α and IL-1β in macrophages and neutrophils. (A, B) Dot plot of cluster-enriched gene expressions. Cell clusters of control kidney (left) and kidneys undergoing UUO (right) were identified by kidney cell-specific marker expression. (C-F) Violin plots of log-transformed gene expression of TNF-α and IL-1β in each cell population (B cell, dentritic cell, distal tubular cell, macrophage, neutrophil, natural killer cell, proximal tubular cell and T cell) of control kidney and kidneys undergoing UUO. (G-J) Comparison of gene expression of TNF-α and IL-1β in macrophages and neutrophils in the kidneys on day 2 and 10 after UUO. *$P < 0.05$; **$P < 0.01$; ***$P < 0.001$.

Supplementary materials

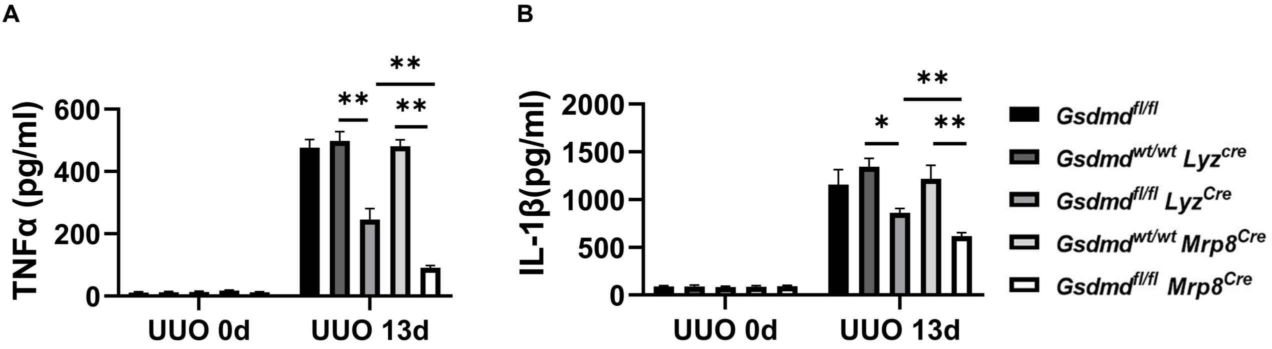

Figure S3. Specific deletion of *Gsdmd* in neutrophils inhibited TNF-α and IL-1β production in kidney after UUO. (A, B) TNF-α and IL-1β production in kidney on day 13 after UUO were detected by ELISA. n=5. *P < 0.05; **P < 0.01.

Supplementary materials

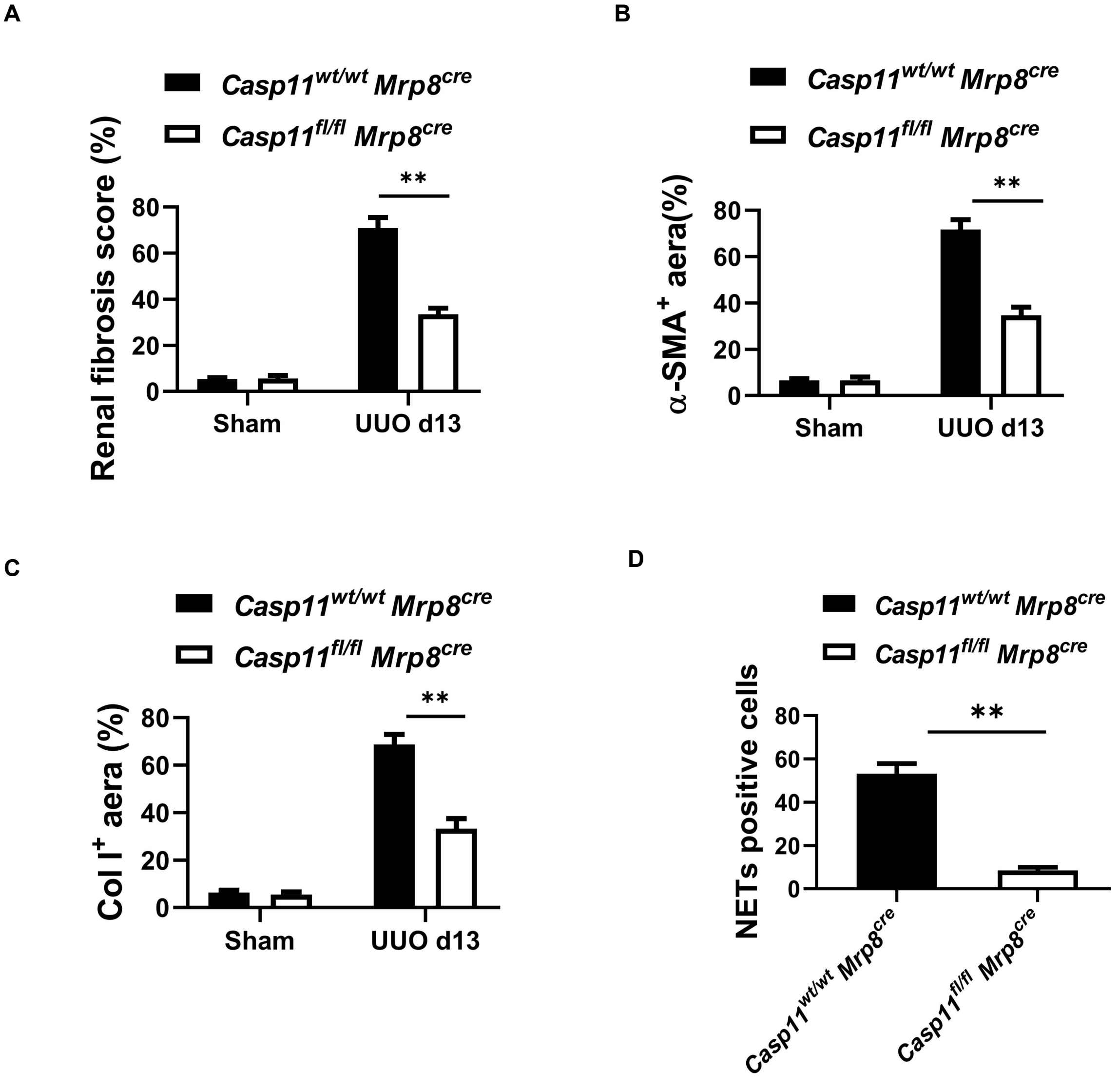

Figure S4. Quantification of renal fibrosis scores, α-SMA and Col I expression and NETs formation after UUO in the kidney of wildtype versus $Casp^{fl/fl}$ $Mrp8^{cre}$ **mice. (A) Quantification of renal fibrosis scores evaluated by Masson trichrome staining. Kidneys were isolated from** $Casp11^{wt/wt}$ $Mrp8^{cre}$ **mice and** $Casp^{fl/fl}$ $Mrp8^{cre}$ **mice on day 13 after UUO. (B, C) Quantification of α-SMA (B) and Col I (C) expression by immunofluorescence. (D) Quantification of cells undergoing NETs. n=6. **$P < 0.01$.**

Supplementary materials

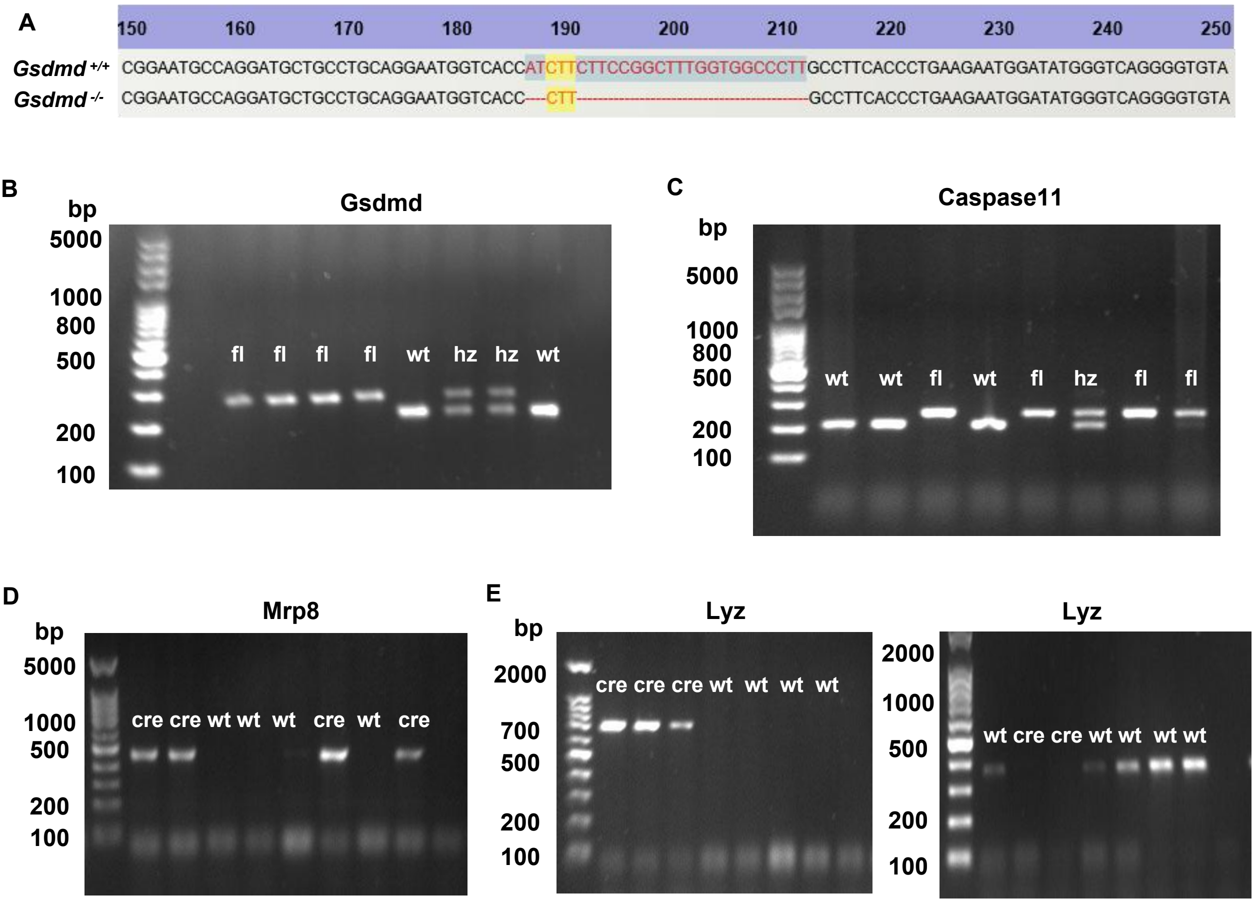

Figure S5. Identification of gene-editing mice. (A) Gene sequences of the targeted region of *Gsdmd*$^{-/-}$ mice. (B) Representative DNA gel of *Gsdmd*$^{fl/fl}$, *Gsdmd*$^{wt/wt}$ mice and heterozygotes. One DNA band is observed for *Gsdmd*$^{fl/fl}$ or *Gsdmd*$^{wt/wt}$ mice, the higher one for *Gsdmd*$^{fl/fl}$ mice and the lower one for *Gsdmd*$^{wt/wt}$ mice. Two DNA bands are observed for heterozygotes (HZ). (C) Representative DNA gel of *Casp11*$^{fl/fl}$, *Casp11*$^{wt/wt}$ mice and heterozygotes. The higher band indicated for *Casp11*$^{fl/fl}$ mice and the lower one indicated for *Casp11*$^{wt/wt}$ mice. Two DNA bands are observed for heterozygotes. (D) Representative DNA gel of *Mrp8-cre* mice. (E) Representative DNA gel of *Lyz-cre* mice and wildtype pups. One DNA band at about 700 bp is observed for *Lyz-cre* mice,while the one at about 350 bp is observed for wildtype pups. If both bands are shown, indicating for heterozygotes.
