# Supplementary material for: GSDMD-dependent neutrophil extracellular traps promote macrophage-to-myofibroblast transition and renal fibrosis in obstructive nephropathy
Source: Cell Death Dis. 2022 Aug 8;13(8):693. doi: 10.1038/s41419-022-05138-4 (PMC9360039; doi:10.1038/s41419-022-05138-4)
Supplement: Supplementary file 4 — manuscript [file 41419_2022_5138_MOESM4_ESM.docx]

**GSDMD-dependent** **neutrophil extracellular traps promote macrophage-to-myofibroblast transition and renal fibrosis in** **obstructive nephropathy**

**Yujia Wang^1,2*^, Yinshuang Li^1,2*^, Zhimin Chen^1,2*^, Ying Yuan^1,2^, Qinglin Su^1,2^, Keng Ye^1,2^, Caiming Chen^1,2^, Guoping Li^3^, Yankun Song^3^, Hong Chen^3🖂^, Yanfang Xu^1,2🖂^**

^1^Department of Nephrology, Blood Purification Research Center, the First Affiliated Hospital, Fujian Medical University, Fuzhou, China.

^2^Fujian Clinical Research Center for Metabolic Chronic Kidney Disease, the First Affiliated Hospital, Fujian Medical University, Fuzhou, China.

^3^Department of Pathology, the First Affiliated Hospital, Fujian Medical University, Fuzhou, China

*****Yujia Wang, Yinshuang Li and Zhimin Chen contributed equally to this work.

**🖂Corresponding author:** Hong Chen, Email: fychenhong@126.com; Yanfang Xu, Email: [xuyanfang99@hotmail.com.](mailto:xuyanfang99@hotmail.com;) Chazhong Road 20, Fuzhou 350005, China. Tel: +86-13600880688; Fax: +86-591-87981028.

**Competing Interests statement**

The authors have declared that no conflict of interest exists.

**Abstract**

Renal fibrosis is a common consequence of various progressive nephropathies, including obstructive nephropathy, and ultimately leads to kidney failure. Infiltration of inflammatory cells is a prominent feature of renal injury after draining blockages from the kidney, and correlates closely with the development of renal fibrosis. However, the underlying molecular mechanism behind the promotion of renal fibrosis by inflammatory cells remains unclear. Herein, we showed that unilateral ureteral obstruction (UUO) induced Gasdermin D (GSDMD) activation in neutrophils, abundant neutrophil extracellular traps (NETs) formation and macrophage-to-myofibroblast transition (MMT) characterized by α-smooth muscle actin (α-SMA) expression in macrophages. *Gsdmd* deletion significantly reduced infiltration of inflammatory cells in the kidneys and inhibited NETs formation, MMT and thereby renal fibrosis. Chimera studies confirmed that *Gsdmd* deletion in bone marrow-derived cells, instead of renal parenchymal cells, provided protection against renal fibrosis. Further, specific deletion of *Gsdmd* in neutrophils instead of macrophages protected the kidney from undergoing fibrosis after UUO. Single-cell RNA sequencing identified robust crosstalk between neutrophils and macrophages. In *vitro*, GSDMD-dependent NETs triggered p65 translocation to the nucleus, which boosted the production of inflammatory cytokines and α-SMA expression in macrophages by activating TGF-β1/Smad pathway. In addition, we demonstrated that caspase-11, that could cleave GSDMD, was required for NETs formation and renal fibrosis after UUO. Collectively, our findings demonstrate that caspase-11/GSDMD-dependent NETs promote renal fibrosis by facilitating inflammation and MMT, therefore highlighting the role and mechanisms of NETs in renal fibrosis.

**Introduction**

Renal fibrosis is the most common outcome of chronic kidney disease (CKD), which is characterized by progressive deposition of the extracellular matrix in renal tissue^1^. Obstructive nephropathy, resulting from ureteral obstruction and eventually leading to renal fibrosis, is a major cause of CKD especially in children^2^. Strategies for protecting kidneys from obstructive injury and preventing the progression of renal insufficiency are lacking. In recent years, executors of different cell death pathways have been confirmed to play a significant part in renal fibrosis^3, 4, 5, 6^. We previously reported that Gasdermin E (GSDME)-mediated pyroptosis in renal parenchymal cells contributes to obstructive nephropathy, while deletion of GSDME in renal tubular cells fails to completely inhibit the development of renal fibrosis^7^. These data present an opportunity to explore the role of other cell types in the pathogenesis of renal fibrosis.

Abundant inflammatory cells are mobilized to infiltrate into the site of kidney injury, followed by an uncontrolled inflammatory response and unchecked production of extracellular matrix (ECM) which ultimately disrupts the architecture of the kidneys^8, 9^. However, the cellular and molecular mechanisms behind the inflammatory responses involved in renal fibrosis after ureteral obstruction are not fully understood. This lack of mechanistic understanding hinders the development of precise clinical measures against renal fibrosis.

Neutrophils serve as front-line cells that mediate inflammatory responses during tissue injury. Moreover, infiltration of neutrophils has been confirmed in kidney injury induced by different means, including ureteral obstruction^7, 10, 11^. Neutrophil extracellular traps (NETs) are extracellular, web-like structures composed of decondensed DNA, histones and neutrophil granule proteins after certain stimuli imposing on neutrophil, through which neutrophil causes tissue damage and interacts with other cells^12^. NETs can play a detrimental role in tissue damage through several mechanisms, including the promotion of vascular occlusion, sterile inflammation, and immune balance disruption^13^. NETs also provide pathways for neutrophils to interact with other immune cells, such as macrophages^14, 15, 16^. The regulation of NETs has been an area of interest in neutrophil biology. Several ground-breaking studies have recently revealed the role of Gasdermin D (GSDMD), an executor of pyroptosis in macrophages and NETs in neutrophils ^17, 18, 19^.

The role of NETs in obstruction-induced renal fibrosis and potential regulatory mechanisms remain unclear. The unilateral ureteral obstruction (UUO) is a widely used model for acute kidney injury and progressive renal fibrosis associated with obstructive nephropathy^20^. In the present study, we found that *Gsdmd* deletion significantly reduced infiltration of inflammatory cells, NETs formation, macrophage-to-myofibroblast transition (MMT) and renal fibrosis. Chimera studies between *Gsdmd^-/-^* and *Gsdmd^+/+^* mice identified that GSDMD from bone marrow-derived cells instead of renal parenchymal cells was involved in renal fibrosis. Interestingly, specific deletion of *Gsdmd* in neutrophils rather than macrophages provided renoprotection for mice after UUO, indicating the key role of GSDMD activation in neutrophils in the process. Single-cell RNA sequencing revealed active crosstalk between neutrophils and macrophages. Studies in *vitro* further determined the promotive effects that NETs imposed on MMT. Furthermore, caspase-11 (Casp11) was revealed to play an important role in NETs formation after UUO. Therefore, our findings show that Casp11/GSDMD-dependent NETs promote inflammation and MMT leading to renal fibrosis in obstructive nephropathy.

**Results**

***Gsdmd* deletion ameliorated renal fibrosis following UUO**

To understand whether *Gsdmd* deficiency affects UUO-induced renal fibrosis, we compared the extent of fibrosis of *Gsdmd^-/-^* and *Gsdmd^+/+^* mice following UUO surgery. Masson Trichrome staining showed obvious collagen deposition in the tubulointerstitial area in *Gsdmd^+/+^* mice on day 13 post-UUO, compared to mice subjected to sham surgery (Fig. 1A-B). Fibrotic markers, including α-smooth muscle actin(α-SMA) and collagen I (Col I), were also upregulated in the kidneys of *Gsdmd^+/+^* mice after UUO (Fig. 1C-G). Overall fibrosis quantified by Masson Trichrome staining and the expression of α-SMA and Col I in kidney were significantly reduced by *Gsdmd* deletion. We also observed UUO-induced activation of kidney GSDMD on day 7 and day 13, as evidenced by the presence of its cleaved form. The knockout efficiency of *Gsdmd* was also confirmed (Fig. 1H). These data suggest that GSDMD contributes to ureteral obstruction-induced renal fibrosis progression.

***Gsdmd* deletion inhibited inflammatory cell infiltration into kidney after UUO**

Inflammation, characterized by monocytes/macrophages and neutrophils infiltration, is concomitant with tubular injury and universally suspected as a culprit in the development of renal fibrosis. We used F4/80 and Ly6G as markers for monocytes/macrophages and neutrophils, respectively. Flow cytometry analyses were performed to compare the percentage of neutrophils and macrophages infiltrating into kidney on day 0, day 2, day 5 and day 10 after UUO between *Gsdmd^-/-^* and *Gsdmd^+/+^* mice. As shown in Fig. 2A-D, *Gsdmd* depletion inhibited neutrophils and macrophages infiltration in the kidney on day 5 and 10 after UUO. The inflammatory cell recruitment emerged in a time-dependent manner after UUO and in the early phase only a small number of inflammatory cells infiltrated in the kidney, which might explain that the effect of *Gsdmd* deletion was not obvious on day 2. The expression of GSDMD was up-regulated following UUO and GSDMD was detected in the interstitial area but not in proximal tubular cells (Fig S1. A-B), indicating the expression of GSDMD in inflammatory cells rather than tubular cells played the predominant role in the development of obstructive nephropathy. Meanwhile, renal expression of proinflammatory and profibrogenic cytokines, including TNF-α, IL-1β, HMGB1 and TGF-β1 remained robust after UUO in *Gsdmd^+/+^* mice. These factors were downregulated in *Gsdmd* null mice (Fig S1.C-F). Immunohistochemistry analyses also confirmed that the number of F4/80^+^ cells and Ly6G^+^ cells increased on day 10 after UUO in the kidneys of *Gsdmd^+/+^* mice, but significantly reduced in *Gsdmd^-/-^* mice (Fig. 2E-H).

**Single-cell sequencing revealed macrophage-to-myofibroblast transition, GSDMD upregulation, NETs activation in neutrophils, and active crosstalk between neutrophils and macrophages**

To investigate the cellular function of neutrophils and macrophages during the process of renal fibrosis, we examined the single-cell transcriptomes of kidneys from mice undergoing UUO after 2 days (2d) and 10 days (10d), as well as control kidneys. Unsupervised clustering analysis identified 11 and 13 distinct cell clusters, respectively (Fig. 3A-C). Cell clusters of control kidney and kidneys undergoing UUO were identified by kidney cell-specific marker expression (Fig S2. A-B). All 11 cell types in UUO-2d were shared with UUO-10d while dendritic and transitional cells were the only two observed unique cell types in UUO-10d. This was consistent with known features of chronicity. The pattern of trajectory for macrophages and myofibroblasts and the heatmap of the top 20 genes significantly changed in pseudotime illustrated a transition from macrophage to myofibroblast (Fig. 3D-F). We observed abundant intercellular signaling between macrophage, neutrophils and myofibroblast (Fig. 3G), thereby supporting the aforementioned findings. We further revealed that *TNF-α* and *IL-1β* as the important inflammatory genes predominantly expressed in macrophages and neutrophils (Fig S2. C-F). Compared to control kidneys, the expression of *TNF-α* and *IL-1β* significantly upregulated on day2 and day10 post-UUO (Fig S2. G-J). These data suggest that macrophages and neutrohils play essential roles in inflammation after UUO.

We further examined *Gsdmd* expression in a variety of healthy and obstruction-injured renal cell types by single-cell sequencing. The expression of *Gsdmd* increased significantly in different immune cell types including neutrophils and macrophages in kidneys after UUO, but very scarce in renal tubular cells (Fig. 3H-K). Up-regulated *Gsdmd* expression in neutrophils remained robust until day 10 after UUO, but that in macrophages on day 10 showed a downtrend compared with day 2. NETs-associated genes in neutrophils were significantly activated by UUO (Fig. 3M), thus, confirming the involvement of NETs formation in obstructive kidneys. Next, we assessed crosstalk between neutrophils and other cell types. We found that macrophage had the most active intercellular crosstalk with neutrophils compared to other immune cells (Fig. 3L). Taken together, we provide solid evidence for up-regulated expression of *Gsdmd* and NETs activation in neutrophil and close interactions between neutrophils and macrophages in the obstructive kidney.

**UUO induced NETs formation in neutrophils and α-SMA production in macrophages, which could be inhibited by *Gsdmd* deletion**

To explore the role of GSDMD in NETs formation, we compared the expression of histone H3 (H3), core component of nucleosome, by immunofluorescence on day 2 and day 7 post-UUO between *Gsdmd^-/-^* and *Gsdmd^+/+^* mice. Myeloperoxidase (MPO) staining was used to confirm neutrophil infiltration. H3 and MPO were stained double positively with loose expansion of chromatin in renal interstitium of *Gsdmd^+/+^* mice after UUO, which was indicative of NETs formation. *Gsdmd* deletion significantly reduced the number of H3 and MPO double positive cells (Fig. 4A-B). Myofibroblasts, a subset of activated fibroblasts, are often characterized using α-SMA as a specific marker^21^. We observed α-SMA expression in F4/80^+^ macrophages on day 7, suggesting MMT in renal fibrosis which was supported by previous studies^22, 23, 24, 25^. The number of cells co-expressing α-SMA and F4/80 in the kidneys of *Gsdmd^-/-^* mice post-UUO were downregulated (Fig. 4C-D). Further, flow cytometry analyses confirmed that *Gsdmd* depletion reduced the percentage of macrophages expressing α-SMA in kidneys on day 7 and 10 after UUO (Fig. 4E-F). These data demonstrate that GSDMD contributes to NETs release from neutrophils and α-SMA expression in macrophages.

***Gsdmd* deletion in bone marrow-derived cells instead of renal parenchymal cells provided protection against renal fibrosis following UUO**

To further examine the contribution of renal parenchymal cells and bone marrow-derived immune cells to the pathogenesis of renal fibrosis post-UUO, we performed chimera studies by bone marrow transplantation. *Gsdmd^+/+^* to *Gsdmd^+/+^* chimeric mice developed severe renal fibrosis post-UUO and displayed increased collagen deposition as determined by Masson Trichrome staining on day 13 after UUO (Fig. 5A, D). These pathological changes were similar in *Gsdmd^+/+^* to *Gsdmd^-/-^* chimeric mice. However, both *Gsdmd^-/-^* to *Gsdmd^+/+^* and *Gsdmd^-/-^* to *Gsdmd^-/-^* chimeric mice showed significant renoprotection. We also observed increased deposition of α-SMA and Col I in the kidneys of *Gsdmd^+/+^* to *Gsdmd^+/+^* and *Gsdmd^+/+^* to *Gsdmd^-/-^* chimeric mice. In contrast, expression of these fibrotic markers was downregulated in *Gsdmd^-/-^* to *Gsdmd^+/+^* and *Gsdmd^-/-^* to *Gsdmd^-/-^* chimeric mice (Fig. 5B, C, E, F). Furthermore, NETs formation was suppressed in *Gsdmd^-/-^* to *Gsdmd^+/+^* chimeras (Fig. 5G-H) as was α-SMA expression in macrophages (Fig. 5I-J) on day 7 after UUO. These data confirm that GSDMD from bone marrow-derived cells, not renal parenchymal cells, is involved in renal fibrosis after UUO.

**Specific deletion of *Gsdmd* in neutrophils instead of macrophages protected the kidney from undergoing fibrosis after UUO**

To distinguish the specific contribution of each inflammatory cell type to prevent fibrosis in the *Gsdmd^-/-^* mouse, we generated neutrophil-specific and macrophage-specific *Gsdmd* deficient mice by crossing *Gsdmd^fl/fl^* mice and with *Mrp8-Cre* or *Lyz-Cre* mice respectively. Compared with wild type mice (*Gsdmd^wt/wt^Mrp8^cre^*), neutrophil-specific *Gsdmd* deficient mice (*Gsdmd^fl/fl^Mrp8^cre^*) significantly reduced renal fibrosis on day 13 after UUO. However, macrophage-specific *Gsdmd* deficient mice (*Gsdmd^fl/fl^Lyz^cre^*) failed to reduce renal fibrosis when compared with wild type mice (*Gsdmd^wt/wt^Lyz^cre^*) (Fig. 6A, D). Consistently, neutrophil-specific *Gsdmd* deficient mice instead of macrophage-specific *Gsdmd* deficient mice reduced the expression of α-SMA (Fig. 6B, E) and Col I (Fig. 6C, F) in the kidneys on day 13 after UUO. We noticed that macrophage-specific *Gsdmd* deletion also inhibited inflammatory cytokines including TNF-α and IL-1β production in kidney (Fig S3.A-B) , suggesting that the excretion of TNF-α and IL-1β in macrophages did not play the leading function in the progression of renal fibrosis induced by ureteral obstruction. Taken together, these data provide evidence that GSDMD activation in neutrophils rather than in macrophages contributes to the progression of renal fibrosis after ureteral obstruction.

**GSDMD-dependent NETs actived p65 translocation into the nucleus in macrophages and promoted inflammation and MMT**

The interaction between neutrophils and macrophages is an interesting area of research and there is growing evidence suggesting NETs as potential pathway through which neutrophils affect macrophages^14, 15^. NETs formation *in vitro* were induced in neutrophils isolated from *Gsdmd^+/+^* and *Gsdmd^-/-^* mice. Low dose (20nM) of phorbol 12-myristate 13-acetate (PMA) was firstly used to prime neutrophils to a low-grade activated state but not to induce NETs formation. Then hydrops extracted from the obstructed kidney and urine of healthy mice were then added to the treatment and control group respectively. Immunofluorescence revealed positive GSDMD expression in *Gsdmd^+/+^* neutrophils in the control and treatment group (Fig. 7A). In addition, we found that GSDMD was primarily localized in the cytoplasm in unstimulated neutrophils. Following hydrops from the obstructed kidney stimulation, GSDMD translocalization to the cell membranes was observed. This stimulation also induced NETs formation characterized by positive Cit-H3 staining in *Gsdmd^+/+^* neutrophils, which was inhibited by *Gsdmd* deletion (Fig. 7B-C). TGF-β1/Smad3 signaling has been recognized as the master regulator of MMT. Significantly, we found that NETs promoted expression of α-SMA and its upstream transcription factor, phosphorylated Smad3, in both *Gsdmd^+/+^* and *Gsdmd^-/-^* macrophages (Fig. 7D-E). Taken together, these data suggest that *Gsdmd* deletion reduces α-SMA expression in macrophages possibly through the NETs signaling pathway. Pre-treatment of macrophages with the known NETs inhibitors, DNase I or neutrophil elastase (NE) inhibitor, prevented α-SMA expression in macrophages (Fig. 7D). Further, treatment with an anti-TGF-β1 antibody prevented α-SMA expression in macrophage stimulated by NETs. This agreed with the hypothesis that NETs could promote MMT through the TGF-β1/Smad3 signaling pathway (Fig. 7D). In addition, NETs promoted the NF-κB p65 subunit translocated into the nucleus of macrophages (Fig. 7F-G). NETs also increased TGF-β1 and TNF-α generation in macrophages, but did not inhibited by *Gsdmd* deletion (Fig. 7H-I). LPS and HMGB1 treatment promoted IL-1β generation in macrophages, which could be inhibited by *Gsdmd* deletion (Fig.7J).

**Caspase 11 activation in neutrophils played an essential role in NETs formation and renal fibrosis after UUO**

It has been well known that GSDMD could be cleaved by caspases including caspase-1, 4, 5 and 11^26^. Previous study revealed that Casp11 was required for GSDMD-dependent NETs formation^18^. In our study, we actually found that Casp11 was activated during the development of obstructive nephropathy (Fig.8A). To examine the role of Casp11 in NETs formation and renal fibrosis after UUO, we generated neutrophil-specific *Casp11* deficient mice by crossing *Casp11^fl/fl^* mice with *Mrp8-Cre* mice. Specific deletion of *Casp11* in neutrophils effectively protected the kidney from undergoing fibrosis (Fig. 8B, Fig S4.A) and expressing α-SMA (Fig. 8C, Fig S4.B) and Col I (Fig. 8D, Fig S4.C) on day 13 after UUO. NETs formation in the kidney induced by ureteral obstruction was significantly reduced by neutrophil-specific deletion of *Casp11* (Fig.8E, Fig S4.D). Consistently, *in vitro*, hydrops extracted from the obstructed kidney induced NETs formation in neutrophils of *WT* mice, which could be inhibited by *Casp11* deletion (Fig. 8F-G). Thus, in response to ureteral obstruction, NETs formation in the renal infiltrating neutrophils depends on Casp11/GSDMD activation.

**Discussion**

As well as contributing to acute phases of kidney injury, leading to more severe tubule damage, immune cell-mediated inflammatory responses also stimulate the transition from AKI to CKD characterized by extracellular matrix deposition and renal fibrosis ^27^. GSDMD, pore-forming protein that executes pyroptosis, has been considered to closely relate with functions of inflammatory cells^28^. We previously revealed that GSDMD-mediated pyroptosis and RIPK3-mediated necroptosis in both myeloid and nonmyeloid cells critically contributed to the progression of sepsis^29^. Herein, we show that bone marrow transplantation from *Gsdmd^-/-^* mice ameliorated renal fibrosis in *Gsdmd^+/+^* mice. However, *Gsdmd^+/+^* to *Gsdmd^-/-^* chimeric mice did not reveal a renoprotective phenotype. Therefore, we reveal the involvement of GSDMD-mediated cellular processes in bone marrow-derived cells, not in renal parenchymal cells, in ureteral obstruction-induced renal fibrosis.

The activation of inflammatory responses and recruitment of leucocytes to an injured kidney come after tubular injury that released chemotactic signals and endothelial injury that enabled the adhesion and transmigration of immune cells^30^. We showed infiltration of a large number of neutrophils and macrophages in the kidneys on day 5 and day 10 after UUO. Significant efforts have been made to explore the mechanism by which tubular injury provokes immune cell infiltration. M. A. Battistone *et al*.^31^ identified a novel chemokine-produced axis between renal tubular cells and intercalated cells, in which uridine diphosphate–glucose, a danger-associated molecular pattern released from damaged tubular cells, could bind to P2Y14 receptors on intercalated cells, followed by chemokine release and proinflammatory immune cell recruitment. H. Maekawa *et al.*^32^ showed that neutrophil infiltration after cisplatin-induced tubular injury was regulated by the mitochondrial damage-cGAS-STING axis. Several well-designed studies showed that CCR2 and CX3CR1 promote monocytes/macrophage infiltration in the kidneys after post-ischemic or post-obstructive injury ^33, 34^.

After migration, neutrophils and macrophages induce inflammation-associated damage via different mechanisms. NETs have been one of the most widely studied pathways of neutrophil-induced injury, which has been observed in kidney injury induced by different stimuli^35^. There is growing interest in the synthesis and regulation of NETs. RIPK1-RIPK3-MLKL-dependent neutrophil necroptosis has previously been associated with NET formation followed by exposure to certain particles^36^ or antineutrophil cytoplasmic antibody (ANCA)^37^. Recent studies proposed a breakthrough hypothesis about the significance of GSDMD in NETs release. S. Kordes *et al.*^17^ demonstrated that NETs formation was dependent on GSDMD, during which GSDMD could be cleavage by neutrophil elastase (NE) and translocated to the plasma membrane to promote NETs release. In addition, GSDMD was shown to play a role in upstream NE mobilization from granules – an essential process for NETs formation, suggesting a feed-forward loop between GSDMD and NE. N. D. Condon *et al.*^18^ revealed caspase-11/GSDMD-dependent mechanism of NETs activation and concomitant cell death (NETosis) as a defensive response against cytosolic bacteria. Caspase-11 and GSDMD were shown to be required for neutrophil plasma membrane rupture during the final stage of NETs extrusion as well as nuclear delobulation and DNA expansion during the early phase. Our study consistently suggested that Casp11/GSDMD were required for NETs formation in the kidneys after ureteral obstruction. More recent findings from A. V. Goncalves *et al.*^19^ showed that GSDMD inhibition prevents multiple organ dysfunction during sepsis by blocking NETs formation. In our study, we determined the suppressive effect of *Gsdmd* deletion on NETs formation in the kidneys after UUO and provided supporting evidence for the vital role of GSDMD in NET formation. As the front-line immune cell at the site of injury, neutrophils may initiate NETs formation in the kidneys as early as 24 h after ischemic injury^38^. In the current study, we observed NETs formation in kidneys on day 7 after ureteral obstruction using Cit-H3 positive staining. This made it possible for neutrophils to take effect in the early injury phase as well as in the later phase oriented towards renal fibrosis.

Interestingly, we discovered that NETs formed by neutrophils imposed a stimulating effect on the TNF-α and TGF-β1 secretion from macrophages by triggering NF-κB p65 subunit translocated into the nucleus, and then promoted MMT through activation of TGF-β1/Smad pathway in macrophages. We identified that specific deletion of *Gsdmd* in neutrophils instead of macrophages prevented the progression of renal fibrosis after UUO, which provided stronger evidence that GSDMD-dependent NETs formation functioned as the upstream mechanism of the activity of macrophages. Previous studies have shown that neutrophils may horn in on macrophage activity by releasing NETs. During atherosclerosis, NETs primed macrophages for cytokine release^16^. NETs stimulated by free fatty acids may influence monocyte-derived macrophage infiltration and inflammation in the liver^39^. In the animal model of post-epidural fibrosis, NETs were found to increase the production of extracellular matrix components in macrophages^40^. In a systemic vasculitis known as deficiency of ADA2, NETs induced production of inflammatory cytokines from macrophages through NF-κB nuclear translocation in macrophages^41^. Macrophages produce large amounts of proinflammatory and profibrotic cytokines which can aggravate tubular damage contributing to renal fibrosis. We observed upregulated expression of cytokines including TNF-α, IL-1β, and TGF-β1 from macrophages in post-obstructive kidneys. These findings were consistent with previous studies from our group ^7^ and others^42, 43^. However, in our *vitro* study, TNF-α and TGF-β1 produced in macrophages could not be prevented by *Gsdmd* deletion. Further investigation is needed to explore subsequent mechanism of how neutrophil extracellular DNA or associated proteins activate TGF-β receptor as well as other inflammatory signals in macrophages. Notably, macrophages are multifunctional cells which exhibit a wide range of complex roles in both renal injury and repair^44^. Homeostatic injury and repair mechanisms in macrophages during kidney injury remain unclear despite considerable efforts.

The transition from macrophage to myofibroblast in the condition of UUO were shown in our study. However, myofibroblasts in kidney fibrosis could originate from different sources besides the MMT program. V. S. LeBleu et al^45^ revealed that about half of myofibroblasts emerged from local resident fibroblasts through proliferation and the rest are recruited through differentiation including MMT (35%), the endothelial-to-mesenchymal transition program (10%) and the epithelial-to-mesenchymal transition program. In the present study, we laid stress on MMT and its upstream regulatory mechanism based on our experimental findings and the effects of targeting diverse pathways to inhibit the accumulation of myofibroblasts after UUO needs to be addressed in further studies.

In conclusion, we found that UUO-induced Casp11/GSDMD-dependent NETs formation in neutrophils activated p65 translocation to the nucleus and then stimulated TNF-α and TGF-β1 production in macrophages, which promoted MMT contributing to renal fibrosis (Fig. 8H). Moreover, conditional deletion of *Gsdmd* in neutrophils effectively prevented renal fibrosis by blocking NETs formation in neutrophils and α-SMA production in macrophages. Therefore, targeting Casp11/GSDMD-dependent NETs in neutrophils could provide a potentially novel therapeutic strategy against progressive obstructive nephropathy.

**Materials and Methods**

**Murine Models**

All the experiments were conducted according to the Chinese Guidelines on the Care and Use of Laboratory Animals, with approval from Laboratory Animal Management and Ethics Committee of Fujian Medical University (FJMU IACUC 2021-0298).

*Gsdmd^-/-^* and *Gsdmd^+/+^* mice on a C57BL/6 background were provided by Prof. Jiahuai Han (State Key Laboratory of Cellular Stress Biology, School of Life Sciences, Xiamen University, China). *Gsdmd^fl/fl^* and *Casp11^em1cyagen^* on a C57BL/6J background were obtained from Cyagen Biosciences. *Lyz2^tm1(cre)Ifo^* and *MRP8-Cre-ires/GFP* mice on a C57BL/6 background were obtained from Jackson Lab. *Gsdmd^fl/fl^* *Lyz^cre^* were generated by intercrossing *Lyz-cre* with *Gsdmd*^fl/fl^ mice. *Gsdmd^fl/fl^Mrp8^cre^* mice were generated by intercrossing *Mrp8-cre* with *Gsdmd*^fl/fl^. *Casp11^fl/f^Mrp8^cree^* mice were generated by intercrossing *Mrp8-cre* with *Casp11^fl/fl^*. *Casp11*^-/-^ mice were obtained by intercrossing the *Casp11^fl/fl^* mice with *EIIa*-*Cre* mice (Jackson Lab, 003724). All mice were housed in a specific pathogen-free facility with 12 h light/dark cycles. Genotypes were determined by tail-snip PCR amplification and the results were shown in FigS5. UUO surgery was performed as previously describe^8^. Briefly, male mice (8–10-weeks-old, 22–26 g body weight) were anesthetized and ureteral obstruction was performed by ligation of the left ureter with 4–0 silk sutures. Sham-operated mice underwent the same procedure without ligation. Kidneys were collected and analyzed at the indicated time points.

For bone marrow transplantation studies, recipient mice were irradiated by lethal X-ray irradiation (7.5 Gy). Subsequently, 2×10^6^ bone marrow cells isolated from donor mice were injected into recipients via the tail vein.

After handling the mice, the investigators were blinded when assessing the outcome (histology, ELISA, Single-cell sequencing and Western blot).

**Reagents and antibodies**

Anti-α-SMA (A5228, Sigma-Aldrich), anti-collagen I (ab34710, Abcam), anti-GSDMD antibody (ab219800, Abcam), anti-pSmad3 antibody (ab52903), anti-Caspase11 (14340, Cell Signaling) and anti-GAPDH (3781, ProSci) antibodies were used for western blotting. Anti-MPO (ab208670, Abcam), Anti-Histone H3 (AF0863, Affinity Bioscience, China), Anti-Histone H3 (citrulline R2+R8+R17) antibody (ab5103, Abcam), anti-F4/80 [CI: A3-1] (ab6640, Abcam), anti-Lrp2/Megalin (ab184676, Abcam), as well as antibodies against α-SMA, collagen I, GSDMD (ab239377), and DAPI were used for immunofluorescence staining. Anti-F4/80 and anti-Ly6G [RB6-8C5] (ab25377, Abcam) antibodies were used for immunohistochemical staining. Mouse TNFα (MTA00B), IL-1β (MLB00C), and TGF-β1 (DB100C) ELISA Kits provided by R&D Systems and HMGB1 (ST51011) ELISA Kit provided by TECAN were used for detection of their secretion levels by enzyme linked immunosorbent assay (ELISA).

**Histologic analysis**

Kidneys samples were fixed in 4% (v/v) normal formalin and embedded in paraffin optimal cutting temperature compound (OCT, 4538, Leica). Paraffin sections (4μm) were stained with Masson Trichrome and with the immunohistochemical stain. Kidney cryosections (4μm) were used for immunofluorescence staining. After fixation with ice-cold acetone for 15 min, sections were washed with phosphate buffered saline (PBS) and incubated with different primary antibodies: anti-α-SMA (1:200), anti-collagen I (1:100), anti-MPO (1:200), anti-Histone H3 (1:200, AF0863), anti-F4/80 (1:100), anti-Lrp2/Megalin (1:200), and anti-GSDMD (1:100) for 1–2 h, followed by staining with Alexa Fluor® 488 and Alexa Fluor® 594 labeled secondary antibodies (Abcam). DNA was labeled with DAPI (Invitrogen). Immunohistochemical staining was performed as previously described ^3^. The numbers of F4/80^+^, Ly6G^+^, NETs^+^, F4/80^+^α-SMA^+^ and GSDMD^+^ cells were accessed with Image J software. The evaluation and scoring of renal fibrosis based on Masson Trichrome staining were performed with published methods^46^. Briefly, after blinded capture of micrographs, each image was evaluated by a technician in a manner blinded to experimental conditions. The collagen deposition in the interstitial area (green) and renal tubules and glomeruli (red) were defined for their intensity values and the total “green” and “red” areas were then determined using Image J software.

**Western blot analysis**

For kidney tissues, approximately 50 mg were placed in 400-500 μL radioimmunoprecipitation assay (RIPA) lysis buffer containing a cocktail of protease inhibitors before homogenization. The Bradford protein assay was used to determine protein concentrations and then sodium dodecyl sulphate (SDS) was added. For cultured cells in vitro, cells were harvested and immediately lysed with 1.2×SDS sample buffer. Lysates were then subjected to electrophoresis and separated by SDS polyacrylamide gel electrophoresis (SDS-PAGE), then transfer to polyvinylidene fluoride membranes (EMD Millipore). Membranes were blocked for 1 h in 5% BSA and incubated overnight at 4°C with the primary antibodies. After TBST washing, membranes were incubated with horseradish peroxidase-labeled secondary antibodies for 1h. The enhanced chemiluminescence (ECL) method was used to visualize the blots.

**Flow cytometry analysis**

Flow cytometry was used to quantify the immune cell subtypes in the injured kidney after UUO. In brief, kidneys were harvested, minced, and incubated with 1 mg/ml type IV collagenase (sigma, 11088858001) and DnaseI (90083, ThermoFisher Scientific,) in DMEM (11965092, Gibco™) for 30 minutes at 37℃in a shaker. The RPMI (31800-089, Gibco™) was added with FBS (085-150, WISENT) to terminate the digestion. The digested kidney tissue suspensions were passed through a mesh with 40μm pore size to remove the undigested tissues and then washed in PBS. 40% and 70% percoll (17-0891-02, GE Healthcare, Sweden) were used for the gradient centrifugation. The final pellets were resuspended in PBS and then loaded to the flow cytometry for analysis.

After blocking nonspecific Fc binding with anti-mouse CD16/32 (14-0161-85, eBioscience™), fresh renal immune cell suspensions were incubated with anti-mouse CD45 antibody (30-F11, 47-0451-82, APC-eFluor™ 780, eBioscience™) to determine total immune cell numbers. Anti-CD45 antibody-labeled samples were labeled with the other following antibodies, anti-mouse F4/80-PE (BM8, 48-4801-82, eBioscience™), and Ly6G/Ly6C (RB6-8C5, 11-5931-82, eBioscience™) antibody to identify macrophages (CD45^+^F4/80^+^) and neutrophils (CD45^+^Ly6G^+^) respectively. Using F4/80-PE (BM8, 48-4801-82, eBioscience™) and α-SMA antibody (53-9760-80, Invitrogen) we identified the expression of F4/80 and α-SMA in macrophages. Subsequent data acquisition was performed by flow cytometer (CytoFLEXB53015, Beckman).

**Single-cell transcriptome sequencing**

Mouse kidney tissues were transferred, minced, and washed according to standard procedure. Tissues were dissociated into single cells using Multi Tissue Dissociation Kit (MACS 130-110-201). Single-cell suspensions were loaded to 10x Chromium for to single cell capture using the 10X Genomics Chromium Single-Cell 3’kit (V3) according to the manufacturer’s instructions. Following cDNA amplification, library construction steps were performed according to the standard protocol. Libraries were sequenced on an Illumina NovaSeq 6000 sequencing system (paired-end multiplexing run,150 bp) by LC-Bio Technology Co. Ltd. (Hangzhou, China).

A total of 8481 cells were isolated and sequenced from control samples, with a mean of 40059 reads and a median of 768 genes detected per cell. In the UUO-d10 sample, we isolated a total of 15235 cells with a mean of 45332 reads and a median of 1261 genes detected per cell. In the UUO-d2 sample, we isolated a total of 20917 cells with a mean of 30222 reads and a median of 1602 genes detected per cell. Sequencing results were demultiplexed and converted to FASTQ format using Illumina bcl2fastq software. Sample demultiplexing, barcode processing and single-cell 3’gene counting were performed using the Cell Ranger pipeline (http://support.10xgenomics.com/single-cell-gene-expression/software/overview/welcome, version 4.0.0). Next, we adapted a workflow that makes of R package Seurat (version 3.1.1). The following parameters were used to filter good quality cells: the total number of expressed genes/cell was >500 and <4000 nGenes; the total number of unique molecular identifier/cell was >500 and <15000 nUMI; exclude cells with high cell complexity (log10GenesPerUMI) ≤0.8; exclude cells with >30% mitochondrial ratio.

All sequences generated in this study have been deposited in the National Center for Biotechnology Gene Expression Omnibus (<https://www.ncbi.nlm.nih.gov/geo/>) with accession number GSE197266.

**Cell culture**

Neutrophils were isolated from bone marrow according to previously published protocols^47^. Briefly, the mice were euthanized using cervical dislocation and doused with 70% ethanol. The bone marrow from the femur and tibia was flushed using a sterile syringe filled with 1× HBSS 0.38% sodium citrate buffer and then collected into a clean 50 mL conical tube. The bone marrow cells were collected by centrifugation at 230 × g for 6 min, then resuspended in 1×HBSS 0.38% sodium citrate buffer. The cell suspension was centrifuged by Percoll gradients. The third (neutrophil) layer was transferred to a clean 15 mL conical tube after the first and second cell layers being removed. The collected neutrophils were added with 1× HBSS 0.38% sodium citrate buffer and resuspended. The purity of the cell population was determined by cytospin staining and cell viability was assessed by dye exclusion.

To stimulate neutrophil to form NETs *in vitro*, neutrophils were treated with very low dose of PMA (20nM) for 3 hours and then treated with hydrops extracted from the obstructed kidney or urine of healthy mice for 8 hours. NETs were identified by the positive staining of anti-Cit-H3 antibody (1:200, abcam5103) and anti-MPO antibody (1:200) as previously described^18^. Briefly, the percentage of neutrophils with positive Cit-H3 was quantified via image analysis of eight to ten nonoverlapping fields of view and deconvolved images were subjected to quantitative analysis with Image J. Total cell number in each field of view was determined by counting DAPI (nuclear). For NETs purification, the culture medium was discarded and neutrophils on the bottom of the culture plate were gently washed with cold PBS. After repeated three times washing, cells undergoing NETs were detached from culture plate and collected in 700 µl of FCS-free medium. Cell debris and NETs fragments were separated by centrifugation at 300 x g 10 minutes at 4°C. The NETs-containing supernatant was transferred to a fresh tube without disrupting the pellet. The concentration were determined by Quant-iT PicoGreen dsDNA Assay Kit (Thermo Fisher, P7589).

Bone marrow derived macrophages (BMDMs) were isolated from the bone marrow of *Gsdmd^+/+^* and *Gsdmd^−/−^* mice. BMDMs were obtained as described ^7^. Differentiated macrophages were seeded in 12-well tissue plate for 24 h and then treated as indicated.

**Statistical analysis**

We performed two replicates for each model in Single-cell transcriptome sequencing. Other results were represent at least three independently performed experiments. Statistical analysis was performed with Prism software (GraphPad Software, Inc.). Unpaired Student’s t tests or two-way analysis of variance test with Bonferroni post-test was used to compare the means of two groups. Bars represent means ± SD. *P*<0.05 was considered to indicate statistical significance.

**Data Availability**

All data needed to evaluate the conclusions in the paper are present in the paper. Additional data related to this paper may be requested from the corresponding author.

**Acknowledgments**

We are grateful to Pro. Jiahuai Han (Xiamen University) for experimental materials. We thank the experimental technical help of Central Laboratory, the First Affiliated Hospital of Fujian Medical University.

**Conflict of Interest**

The authors declare no competing interests.

**Author contributions**

HC and YX designed research. YW, YL, ZC, YY, QS, KY, CC, GL,YS, HC and YX performed the experiments. YW, YL, ZC and YX analyzed the data. YW wrote the draft manuscript. HC and YX supervised the research. YX performed writing-review and editing. All authors verified the data and approved the final version of the manuscript.

**Funding**

This work was supported by National Natural Science Foundation of China (No. 82070720), Natural Science Foundation of Fujian province (No.2020J02020, No. 2021J05151), Fujian Provincial Health Technology Project (No. 2021ZQNZD004), and Fujian Province Finance Project (2020B009). Yanfang Xu was supported by Outstanding Young Talents Program of the First Affiliated Hospital of Fujian Medical University (YJCQN-A-XYF2021).

**Ethics approval and consent to participate**

The animal experiments were accomplished in compliance with ethical standards. All the animal experiments were performed with the approval of the Laboratory Animal Management and Ethics Committee of Fujian Medical University, according to the Chinese Guidelines on the Care and Use of Laboratory Animals.

**Reference**

1. Nastase MV, Zeng-Brouwers J, Wygrecka M, Schaefer L. Targeting renal fibrosis: Mechanisms and drug delivery systems. *Adv Drug Deliv Rev* 2018, **129:** 295-307.

2. McLeod DJ, Sebastiao YV, Ching CB, Greenberg JH, Furth SL, Becknell B. Longitudinal kidney injury biomarker trajectories in children with obstructive uropathy. *Pediatr Nephrol* 2020, **35**(10)**:** 1907-1914.

3. Chen H, Fang Y, Wu J, Chen H, Zou Z, Zhang X*, et al.* RIPK3-MLKL-mediated necroinflammation contributes to AKI progression to CKD. *Cell Death Dis* 2018, **9**(9)**:** 878.

4. Imamura M, Moon JS, Chung KP, Nakahira K, Muthukumar T, Shingarev R*, et al.* RIPK3 promotes kidney fibrosis via AKT-dependent ATP citrate lyase. *JCI Insight* 2018, **3**(3).

5. Belavgeni A, Meyer C, Stumpf J, Hugo C, Linkermann A. Ferroptosis and Necroptosis in the Kidney. *Cell Chem Biol* 2020, **27**(4)**:** 448-462.

6. Maremonti F, Meyer C, Linkermann A. Mechanisms and Models of Kidney Tubular Necrosis and Nephron Loss. *J Am Soc Nephrol* 2022.

7. Li Y, Yuan Y, Huang ZX, Chen H, Lan R, Wang Z*, et al.* GSDME-mediated pyroptosis promotes inflammation and fibrosis in obstructive nephropathy. *Cell Death Differ* 2021.

8. Ding Y, Kim S, Lee SY, Koo JK, Wang Z, Choi ME. Autophagy regulates TGF-beta expression and suppresses kidney fibrosis induced by unilateral ureteral obstruction. *J Am Soc Nephrol* 2014, **25**(12)**:** 2835-2846.

9. Truong LD, Petrusevska G, Yang G, Gurpinar T, Shappell S, Lechago J*, et al.* Cell apoptosis and proliferation in experimental chronic obstructive uropathy. *Kidney Int* 1996, **50**(1)**:** 200-207.

10. Raup-Konsavage WM, Wang Y, Wang WW, Feliers D, Ruan H, Reeves WB. Neutrophil peptidyl arginine deiminase-4 has a pivotal role in ischemia/reperfusion-induced acute kidney injury. *Kidney Int* 2018, **93**(2)**:** 365-374.

11. Maekawa H, Inoue T, Ouchi H, Jao TM, Inoue R, Nishi H*, et al.* Mitochondrial Damage Causes Inflammation via cGAS-STING Signaling in Acute Kidney Injury. *Cell Rep* 2019, **29**(5)**:** 1261-1273.e1266.

12. Sorensen OE, Borregaard N. Neutrophil extracellular traps - the dark side of neutrophils. *J Clin Invest* 2016, **126**(5)**:** 1612-1620.

13. Papayannopoulos V. Neutrophil extracellular traps in immunity and disease. *Nat Rev Immunol* 2018, **18**(2)**:** 134-147.

14. Josefs T, Barrett TJ, Brown EJ, Quezada A, Wu X, Voisin M*, et al.* Neutrophil extracellular traps promote macrophage inflammation and impair atherosclerosis resolution in diabetic mice. *JCI Insight* 2020, **5**(7).

15. Chen L, Zhao Y, Lai D, Zhang P, Yang Y, Li Y*, et al.* Neutrophil extracellular traps promote macrophage pyroptosis in sepsis. *Cell Death Dis* 2018, **9**(6)**:** 597.

16. Warnatsch A, Ioannou M, Wang Q, Papayannopoulos V. Inflammation. Neutrophil extracellular traps license macrophages for cytokine production in atherosclerosis. *Science* 2015, **349**(6245)**:** 316-320.

17. Sollberger G, Choidas A, Burn GL, Habenberger P, Di Lucrezia R, Kordes S*, et al.* Gasdermin D plays a vital role in the generation of neutrophil extracellular traps. *Sci Immunol* 2018, **3**(26).

18. Chen KW, Monteleone M, Boucher D, Sollberger G, Ramnath D, Condon ND*, et al.* Noncanonical inflammasome signaling elicits gasdermin D-dependent neutrophil extracellular traps. *Sci Immunol* 2018, **3**(26).

19. Silva CM, Wanderley CWS, Veras FP, Sonego F, Nascimento DC, Goncalves AV*, et al.* Gasdermin D inhibition prevents multiple organ dysfunction during sepsis by blocking NET formation. *Blood* 2021.

20. Chevalier RL, Forbes MS, Thornhill BA. Ureteral obstruction as a model of renal interstitial fibrosis and obstructive nephropathy. *Kidney Int* 2009, **75**(11)**:** 1145-1152.

21. Tang PM, Nikolic-Paterson DJ, Lan HY. Macrophages: versatile players in renal inflammation and fibrosis. *Nat Rev Nephrol* 2019, **15**(3)**:** 144-158.

22. Wang YY, Jiang H, Pan J, Huang XR, Wang YC, Huang HF*, et al.* Macrophage-to-Myofibroblast Transition Contributes to Interstitial Fibrosis in Chronic Renal Allograft Injury. *J Am Soc Nephrol* 2017, **28**(7)**:** 2053-2067.

23. Tang PM, Zhang YY, Xiao J, Tang PC, Chung JY, Li J*, et al.* Neural transcription factor Pou4f1 promotes renal fibrosis via macrophage-myofibroblast transition. *Proc Natl Acad Sci U S A* 2020, **117**(34)**:** 20741-20752.

24. Tang PM, Zhou S, Li CJ, Liao J, Xiao J, Wang QM*, et al.* The proto-oncogene tyrosine protein kinase Src is essential for macrophage-myofibroblast transition during renal scarring. *Kidney Int* 2018, **93**(1)**:** 173-187.

25. Meng XM, Wang S, Huang XR, Yang C, Xiao J, Zhang Y*, et al.* Inflammatory macrophages can transdifferentiate into myofibroblasts during renal fibrosis. *Cell Death Dis* 2016, **7**(12)**:** e2495.

26. Wang K, Sun Q, Zhong X, Zeng M, Zeng H, Shi X*, et al.* Structural Mechanism for GSDMD Targeting by Autoprocessed Caspases in Pyroptosis. *Cell* 2020, **180**(5)**:** 941-955 e920.

27. Zuk A, Bonventre JV. Acute Kidney Injury. *Annu Rev Med* 2016, **67:** 293-307.

28. Demarco B, Grayczyk JP, Bjanes E, Le Roy D, Tonnus W, Assenmacher CA*, et al.* Caspase-8-dependent gasdermin D cleavage promotes antimicrobial defense but confers susceptibility to TNF-induced lethality. *Sci Adv* 2020, **6**(47).

29. Chen H, Li Y, Wu J, Li G, Tao X, Lai K*, et al.* RIPK3 collaborates with GSDMD to drive tissue injury in lethal polymicrobial sepsis. *Cell Death Differ* 2020, **27**(9)**:** 2568-2585.

30. Ferenbach DA, Bonventre JV. Mechanisms of maladaptive repair after AKI leading to accelerated kidney ageing and CKD. *Nat Rev Nephrol* 2015, **11**(5)**:** 264-276.

31. Battistone MA, Mendelsohn AC, Spallanzani RG, Allegretti AS, Liberman RN, Sesma J*, et al.* Proinflammatory P2Y14 receptor inhibition protects against ischemic acute kidney injury in mice. *J Clin Invest* 2020, **130**(7)**:** 3734-3749.

32. Maekawa H, Inoue T, Ouchi H, Jao TM, Inoue R, Nishi H*, et al.* Mitochondrial Damage Causes Inflammation via cGAS-STING Signaling in Acute Kidney Injury. *Cell Rep* 2019, **29**(5)**:** 1261-1273 e1266.

33. Li L, Huang L, Sung SS, Vergis AL, Rosin DL, Rose CE, Jr.*, et al.* The chemokine receptors CCR2 and CX3CR1 mediate monocyte/macrophage trafficking in kidney ischemia-reperfusion injury. *Kidney Int* 2008, **74**(12)**:** 1526-1537.

34. Peng X, Zhang J, Xiao Z, Dong Y, Du J. CX3CL1-CX3CR1 Interaction Increases the Population of Ly6C(-)CX3CR1(hi) Macrophages Contributing to Unilateral Ureteral Obstruction-Induced Fibrosis. *J Immunol* 2015, **195**(6)**:** 2797-2805.

35. Nakazawa D, Marschner JA, Platen L, Anders HJ. Extracellular traps in kidney disease. *Kidney Int* 2018, **94**(6)**:** 1087-1098.

36. Desai J, Foresto-Neto O, Honarpisheh M, Steiger S, Nakazawa D, Popper B*, et al.* Particles of different sizes and shapes induce neutrophil necroptosis followed by the release of neutrophil extracellular trap-like chromatin. *Sci Rep* 2017, **7**(1)**:** 15003.

37. Schreiber A, Rousselle A, Becker JU, von Massenhausen A, Linkermann A, Kettritz R. Necroptosis controls NET generation and mediates complement activation, endothelial damage, and autoimmune vasculitis. *Proc Natl Acad Sci U S A* 2017, **114**(45)**:** E9618-E9625.

38. Nakazawa D, Kumar SV, Marschner J, Desai J, Holderied A, Rath L*, et al.* Histones and Neutrophil Extracellular Traps Enhance Tubular Necrosis and Remote Organ Injury in Ischemic AKI. *J Am Soc Nephrol* 2017, **28**(6)**:** 1753-1768.

39. van der Windt DJ, Sud V, Zhang H, Varley PR, Goswami J, Yazdani HO*, et al.* Neutrophil extracellular traps promote inflammation and development of hepatocellular carcinoma in nonalcoholic steatohepatitis. *Hepatology* 2018, **68**(4)**:** 1347-1360.

40. Jin Z, Sun J, Song Z, Chen K, Nicolas YSM, Kc R*, et al.* Neutrophil extracellular traps promote scar formation in post-epidural fibrosis. *NPJ Regen Med* 2020, **5**(1)**:** 19.

41. Carmona-Rivera C, Khaznadar SS, Shwin KW, Irizarry-Caro JA, O'Neil LJ, Liu Y*, et al.* Deficiency of adenosine deaminase 2 triggers adenosine-mediated NETosis and TNF production in patients with DADA2. *Blood* 2019, **134**(4)**:** 395-406.

42. Chen W, Yuan H, Cao W, Wang T, Chen W, Yu H*, et al.* Blocking interleukin-6 trans-signaling protects against renal fibrosis by suppressing STAT3 activation. *Theranostics* 2019, **9**(14)**:** 3980-3991.

43. Misseri R, Meldrum DR, Dinarello CA, Dagher P, Hile KL, Rink RC*, et al.* TNF-alpha mediates obstruction-induced renal tubular cell apoptosis and proapoptotic signaling. *Am J Physiol Renal Physiol* 2005, **288**(2)**:** F406-411.

44. Huen SC, Cantley LG. Macrophages in Renal Injury and Repair. *Annu Rev Physiol* 2017, **79:** 449-469.

45. LeBleu VS, Taduri G, O'Connell J, Teng Y, Cooke VG, Woda C*, et al.* Origin and function of myofibroblasts in kidney fibrosis. *Nat Med* 2013, **19**(8)**:** 1047-1053.

46. Rutkowski JM, Wang ZV, Park AS, Zhang J, Zhang D, Hu MC*, et al.* Adiponectin promotes functional recovery after podocyte ablation. *J Am Soc Nephrol* 2013, **24**(2)**:** 268-282.

47. Ubags NDJ, Suratt BT. Isolation and Characterization of Mouse Neutrophils. *Methods Mol Biol* 2018, **1809:** 45-57.

**Figure legends**

**Fig. 1 Mice with *Gsdmd* deficiency had ameliorated renal fibrosis after UUO. (A)** Representative images of Masson trichrome staining of kidney sections. Kidneys were isolated from *Gsdmd^+/+^* mice and *Gsdmd^−/−^* mice on day 13 after UUO. Scale bar: 100μm. **(B)** Quantification of renal fibrosis scores evaluated by Masson trichrome staining. n=6. **(C, E)** Representative images of immunofluorescence staining of kidney sections, showing the expression of α-smooth muscle actin (α-SMA) (C) and type-I collagen (Col I) (E). DAPI was used for nuclear staining. Scale bar: 100μm. **(D, F)** Quantification of α-SMA (D) and Col I (F) expression by immunofluorescence. n=6. **(G)** Western blot analysis for α-SMA and Col I expression of kidney tissue lysates, which were isolated on day 7 and 13 after UUO. GAPDH was used as a loading control. n=4. **(H)** Western blot showing GSDMD cleavage in kidney after UUO. Kidney tissue lysates were isolated on day 0, 7 and 13 after UUO. The knockout efficiency of *Gsdmd* was also confirmed, and the cleaved GSDMD-N could be observed in WT kidneys. n=4. ***P* < 0.01.

**Fig. 2 Ablation of *Gsdmd* reduced the infiltration of neutrophils and macrophages in the kidneys after UUO. (A, C)** Flow cytometry was used to evaluate the cellular dynamics of neutrophils (Ly6G^+^) and macrophages (F4/80^+^) infiltration in the kidneys of *Gsdmd^+/+^* mice and *Gsdmd^−/−^* mice on day 0, 2, 5 and 10 after UUO. Representative images of flow cytometry analysis were shown in A and C. **(B, D)** Quantification of the percentage of neutrophils (Ly6G^+^) and macrophages (F4/80^+^) infiltration. n=3. **(E, G)** Representative images of immunohistochemistry staining with F4/80^+^ (E) and Ly6G^+^ (G). Scale bar: 100μm. **(F, H)** Quantification of the number of F4/80^+^ (F) and Ly6G^+^ (H) cells per hpf. n=5. ***P* < 0.01.

**Fig. 3 Single-cell sequencing showed macrophage-to-myofibroblast transition, GSDMD upregulation, NETs activation in neutrophils and activated crosstalk between neutrophils and macrophages in the obstructive kidneys. (A-C)** t-distributed stochastic neighbor embedding (tSNE) plot of cell clusters identified on the basis of the expression of highly variable genes in the kidneys from control group (n=2), obstructive kidney on day 2 (n=2) and day 10 (n=2) after UUO. tSNE analysis showed 11 distinct clusters of renal cells in control (A) and 13 distinct clusters in UUO (C) mice. The distribution of cell clusters was consistent between obstructive kidney on day 2 and day 10 (B). DCTs: distal convoluted tubules; ECs: endothelial cells; Fibro: fibroblast; Macro: macrophage; Myofibro: myofibroblast; Neutro: neutrophil; NK: natural killer cell; Podo: podocyte; PTCs: proximal tubular cells; DC: dentritic cell; TE: transitional epithelia. **(D, E)** Unsupervised trajectory of macrophage-to-myofibroblast transition along pseudotime using Monocle. **(F)** Heatmap of top 20 genes significantly changed in macrophage-to-myofibroblast transition in pseudotime. Each row represents a gene. The left and right end corresponds to the starting point (macrophage) and the ending point (myofibroblast) respectively. Color scheme represents the z-score distribution from -3.0 to 3.0. **(G)** Network visualization of the ligands expressed by macrophage and the other cells expressing the cognate receptors primed to receive the signal. Numbers indicate the quantity of ligand-receptor pairs for each intercellular crosstalk. **(H, I)** Violin plots of log-transformed gene expression of GSDMD in each cell population of control kidney (H) and obstructive kidney on day 2 and 10 after UUO (I). **(J, K)** Comparison of gene expression of GSDMD in neutrophils (J) and macrophages (K) between control kidney and obstructive kidney on day 2 and 10 after UUO. **(L)** Network visualization of the ligands expressed by neutrophil and the other cells expressing the cognate receptors primed to receive the signal. Numbers indicate the quantity of ligand-receptor pairs for each intercellular crosstalk. **(M)** Heat map showing the difference of NETs-associated genes expression in neutrophils among control kidney and obstructive kidney on day 2 and 10 after UUO. The colors of yellow to blue represented alterations from high expression to low expression. **P* < 0.05; ****P* < 0.001.

**Fig. 4** **Deletion of *Gsdmd* suppressed NETs formation and α-SMA expression in macrophages in kidney after UUO. (A)** Representative images of immunofluorescence staining of kidney sections, showing the expression of Histone-H3 and MPO. Kidneys were isolated from *Gsdmd^+/+^* mice and *Gsdmd^−/−^* mice on day 2 and 7 after UUO. Scale bar: 100μm. **(B)** Quantification of cells undergoing NETs formation characterized by Histone-H3 and MPO double positive. n=6. **(C)** Representative images of immunofluorescence staining of kidney sections, showing the coexpression of α-SMA and F4/80 in macrophage-to-myofibroblast trasition cells on day 7 after UUO. Scale bar: 100μm. **(D)** Quantification of cells coexpressing α-SMA and F4/80 by immunofluorescence. n=6. **(E)** Flow cytometry was used to evaluate the percentage of macrophages expressing α-SMA on day 0, 7 and 10 after UUO in the kidneys of *Gsdmd^+/+^* mice and *Gsdmd^−/−^* mice. **(F)** Quantification of the percentage of macrophages expressing α-SMA in the kidneys by flow cytometry analysis. n=3. ***P* < 0.01.

**Fig. 5 *Gsdmd* deficiency in bone marrow-derived cells instead of renal parenchymal cells reduced renal fibrosis progression after UUO. (A)** Representative images of Masson trichrome staining of kidney sections. Kidneys were isolated from bone marrow chimeric mice (*Gsdmd^+/+^* to *Gsdmd^+/+^*, *Gsdmd^+/+^* to *Gsdmd^-/-^*, *Gsdmd^-/-^* to *Gsdmd^+/+^* and *Gsdmd^-/-^* to *Gsdmd^-/-^*) on day 13 after UUO. Scale bar: 100μm. **(B, C)** Representative images of immunofluorescence staining of kidney sections from bone marrow chimeric mice, showing the expression of α-SMA (B) and Col I (C). DAPI (blue) was used for nuclear staining. Scale bar: 100μm. **(D-F)** Quantification of renal fibrosis scores evaluated by Masson trichrome staining (D), α-SMA (E) and Col I (F) expression by immunofluorescence. n=6. **(G, H)** Representative images of immunofluorescence staining of kidney sections from *Gsdmd^+/+^* to *Gsdmd^-/-^*, *Gsdmd^-/-^* to *Gsdmd^+/+^* mice on day 7 after UUO, showing the expression of Histone-H3 and MPO and quantification of cells undergoing NETs formation characterized by H3 and MPO double positive. n=6. Scale bar: 100μm. **(I, J)** Representative images of immunofluorescence staining of kidney sections from *Gsdmd^+/+^* to *Gsdmd^-/-^*, *Gsdmd^-/-^* to *Gsdmd^+/+^* mice, showing the coexpression of α-SMA and F4/80 in macrophage-to-myofibroblast trasition cells on day 7 after UUO and quantification of cells coexpressing α-SMA and F4/80 by immunofluorescence. n=6. Scale bar: 100μm. **P* < 0.05; ***P* < 0.01; ^##^*P* < 0.01.

**Fig. 6 Specific deletion of *Gsdmd* in neutrophils instead of macrophages protected the kidney from undergoing fibrosis after UUO. (A)** Representative images of Masson trichrome staining of kidney sections. Kidneys were collected from *Gsdmd^fl/fl^ , Gsdmd^wt/wt^Lyz^cre^, Gsdmd^fl/fl^Lyz^cre^, Gsdmd^wt/wt^Mpr8^cre^* and *Gsdmd^fl/fl^Mpr8^cre^* mice on day 13 after UUO. Scale bar: 100μm. **(B, C)** Representative images of immunofluorescence staining of kidney sections, showing the expression of α-SMA (B) and Col I (C). DAPI (blue) was used for nuclear staining. Scale bar: 100μm. **(D-F)** Quantification of renal fibrosis scores evaluated by Masson trichrome staining (D), α-SMA (E) and Col I (F) expression by immunofluorescence. n=6. ***P* < 0.01.

**Fig. 7 In *vitro* studies revealed that NETs promoted macrophage-to-myofibroblast transition.** **(A)** Representative images of immunofluorescence staining of GSDMD (green). The neutrophils were isolated from the bone marrow of wild-type mice. Neutrophils were primed with very low dose of PMA 20 nM for 3 hours and then treated with hydrops extracted from the obstructed kidney (treatment group) or urine of healthy mice (control group) for 8 hours. GSDMD translocalization to the cell membranes was induced in the treatment group. Scale bar: 50μm. n=4. **(B, C)** Representative images of immunofluorescence staining of Cit-H3 and MPO and quantification of neutrophils undergoing NETs formation characterized by Cit-H3 positive. The neutrophils were isolated from the bone marrow of *Gsdmd^+/+^* mice and *Gsdmd^−/−^* mice and treated with as A described. n=6. Scale bar: 50μm. **(D)** Representative images of immunofluorescence staining of the expression of α-SMA. The macrophages were treated with NETs, NETs plus DNase I(10 U/ml), NETs plus NE inhibitor (Alvelestat, 10μmol/ml) or NETs plus antiTGF-β1 antibody for 96h. Scale bar: 50μm. **(E)** Western blot analysis for α-SMA and phosphorylated Smad3 expression of macrophages, which was corresponding to the five condition of (D). n=4. **(F, G)** Representative images of immunofluorescence staining of p65. Macrophages were treated with NETs, or NETs plus DNase I. Scale bar: 50μm. **(H, I)** Macrophages were treated as described in (D). TNF-α and TGF-β1 production in macrophages were detected by ELISA. n=4. **(J)** Macrophages were treated with 1μg/mL LPS and 500 ng/ml HMGB1 for 18 h. IL-1β production in macrophages were detected by ELISA. The macrophages were isolated from the bone marrow of *Gsdmd^+/+^* mice and *Gsdmd^−/−^* mice and treated with HMGB1 or not. n=4.** *P* < 0.01.

**Fig. 8 Specific deletion of *Casp11* in neutrophils inhibited NETs formation and renal fibrosis after UUO. (A)** Western blot showing caspase11 activation in the kidneys after UUO. Kidney tissue lysates were isolated on day 0, 2, 5 and 10 after UUO. n=4. **(B)** Representative images of Masson trichrome staining of kidney sections from *Casp11^wt/wt^Mrp8^cre^* and *Casp11^fl/fl^Mrp8^cre^* mice on day 13 after UUO. Scale bar: 100μm. n=6. **(C, D)** Representative images of immunofluorescence staining of kidney sections from *Casp11^wt/wt^Mrp8^cre^* and *Casp11^fl/fl^Mrp8^cre^* mice, showing the expression of α-SMA (C) and Col I (D). DAPI (blue) was used for nuclear staining. Scale bar: 100μm. **(E)** Representative images of immunofluorescence staining of kidney sections from *Casp11^wt/wt^Mrp8^cre^* and *Casp11^fl/fl^Mrp8^cre^* mice on day 10 after UUO, showing the expression of Histone-H3 and MPO. Scale bar: 100μm. **(F, G)** Representative images of immunofluorescence staining of Cit-H3 and MPO and quantification of neutrophils undergoing NETs formation. The neutrophils were isolated from *Casp11^+/+^* and *Casp11^-/-^* mice and treated as described in Figure 7A. n=6. Scale bar: 50μm. (**H)** Schematic model of GSDMD-dependent NETs promoting MMT and renal fibrosis in obstructive nephropathy.
